# Chronic NOS Inhibition Affects Oxidative State and Antioxidant Response Differently in the Kidneys of Young Normotensive and Hypertensive Rats

**DOI:** 10.1155/2019/5349398

**Published:** 2019-11-22

**Authors:** M. Majzunova, M. Kvandova, A. Berenyiova, P. Balis, I. Dovinova, S. Cacanyiova

**Affiliations:** ^1^Institute of Normal and Pathological Physiology, Centre of Experimental Medicine, Slovak Academy of Sciences, Bratislava, Slovakia; ^2^Department of Animal Physiology and Ethology, Faculty of Natural Sciences, Comenius University, Bratislava, Slovakia; ^3^Department of Cardiology 1, Center for Cardiology, Universitätsmedizin Mainz, Mainz, Germany

## Abstract

Deficiency of nitric oxide (NO) and oxidative stress can be a cause, a consequence, or, more often, a potentiating factor for hypertension and hypertensive renal disease. Both NO and superoxide anions are radical molecules that interact with each other, leading to oxidative damage of such organs as the kidney. In the present study, we investigated the effect of chronic-specific (neuronal NOS inhibition) and nonspecific NOS inhibition on the oxidative state and antioxidant response and associated oxidative damage of the kidney of young normotensive and hypertensive rats. Young male normotensive Wistar rats (WRs, age 4 weeks) and spontaneously hypertensive rats (SHRs, age 4 weeks) were divided into three groups for each strain by the type of administered compounds. The first group was treated with 7-nitroindazole (WR+7-NI; SHR+7-NI), the second group was treated with N(G)-nitro-L-arginine-methyl ester (WR+L-NAME; SHR+L-NAME), and the control group was treated with pure drinking water (WR; SHR) continuously for up to 6 weeks. Systolic blood pressure increased in WR+L-NAME after the first week of administration and increased slightly in SHR+L-NAME in the third week of treatment. 7-NI had no effect on blood pressure. While total NOS activity was not affected by chronic NOS inhibition in any of the WR groups, it was attenuated in SHR+7-NI and SHR+L-NAME. Nitration of proteins (3-nitrotyrosine expression) was significantly reduced in WR+7NI but not in WR+L-NAME and increased in SHR+7-NI and SHR+L-NAME. Immunoblotting analysis of SOD isoforms showed decreased SOD2 and SOD3 expressions in both WR+7-NI and WR+L-NAME followed by increased SOD activity in WR+L-NAME. Conversely, increased expression of SOD2 and SOD3 was observed in SHR+L-NAME and SHR+7-NI, respectively. SOD1 expression and total activity of SOD did not change in the SHR groups. Our results show that the antioxidant defense system plays an important role in maintaining the oxidative state during NO deficiency. While the functioning antioxidant system seeks to balance the oxidation state in the renal cortex of normotensive WRs, the impaired antioxidant activity leads to the development of oxidative damage of proteins in the kidney induced by peroxynitrite in SHRs.

## 1. Introduction

Nitric oxide (NO), the main vasodilator molecule in the cardiovascular system, is a critical factor in the overall regulation of blood pressure and renal function. At the level of the kidney, NO regulates renal hemodynamics, pressure natriuresis, tubular sodium transport, tubuloglomerular feedback, and renal sympathetic nerve effects and renin release. In the kidney, 3 isoforms of nitric oxide synthase (NOS) are responsible for NO production: neuronal NOS (nNOS), endothelial NOS (eNOS), and inducible NOS (iNOS) [1].

The decreased bioavailability of NO may lead to impaired renal function and consequently may affect the development of hypertension. One of the reasons for the reduced bioavailability of NO is the rapid reaction of NO and superoxide anions followed by the formation of peroxynitrite (ONOO-) [2]. ONOO- can induce eNOS uncoupling, where uncoupled NOS produces additional superoxide instead of NO. Peroxynitrite is cytotoxic and reacts with various biological molecules by mechanism S-nitrosylation. Nitration of protein tyrosine can affect protein function and oxidatively damage lipids and DNA [3, 4], which can lead to organ damage and subsequent development of hypertension [5, 6]. The major source of reactive oxygen species (ROS) in the kidney is NADPH oxidases (Nox). Nox4 is the predominant isoform expressed in the renal cortex [7, 8], and the increased production of ROS observed in 3-month-old SHRs in the kidney might be explained by the upregulation of Nox4 and downregulation of the antioxidant response [9]. N(G)-nitro-L-arginine-methyl ester (L-NAME)-induced NOS inhibition increases kidney Nox activity and Nox4 protein expression but does not affect the Nrf2 transcription factor regulating the antioxidant response in Wistar rats [10]. Angiotensin II (Ang II) can activate Nox, which may trigger another sources of radicals [6]. Similarly, increased activity of renin-angiotensin system (RAS) was observed in hypertensive animals, which may be affected by NO produced in the kidney [3, 11] and can lead to the elevated production of ROS. The presence of ROS stimulates the antioxidant response, which ensures the redox balance. Major endogenous antioxidants are superoxide dismutases (SOD) characterized by different locations: cytosolic SOD (SOD1), mitochondrial SOD (SOD2), and extracellular SOD (SOD3). All three SOD isoforms are generally found in the kidney, where they catalyze dismutation superoxide to hydrogen peroxide [5, 12]. Although the SOD1 isoform represents up to 80% of the total SOD activity in the mammalian kidney, SOD2 appears to be more important in the pathogenesis of kidney disease [5, 13, 14]. In hypertensive animals, the antioxidant response is often disrupted, leading to oxidative stress followed by the development of end-organ damage in such organs as the kidney [9].

The contribution of NO deficiency and oxidative stress in kidney diseases and the diseases affecting kidney functions, including hypertension, has not been determined. For a closer look at the effect of NO deficiency on the redox state in the kidney, we used two different inhibitors of NOS, N^G^-nitro-L-arginine-methyl ester (L-NAME) and 7-nitroindazole (7-NI). Short- and long-term administrations of L-NAME (nonselective inhibitor of NOS) in moderate and high doses cause persistent hypertension associated with multiple renal functional and morphological damage [15–17]. Studies show that the L-NAME-induced inhibition of NOS can lead to stimulation of RAS [18–20] and may result in an increase of ROS production [21, 22]. Oxidative injury of the kidney present in L-NAME-treated normotensive rats is probably mediated by Ang II. Chronic L-NAME-induced inhibition of NOS decreases the activity of SOD and glutathione peroxidase but stimulates catalase activity in the kidneys of adult normotensive rats [23, 24]. In young SHR, significantly reduced dimethylarginine dimethylaminohydrolase (DDAH) activity and increased renal oxidative damage were observed after long-term administration of L-NAME [25]. However, the effects of NOS inhibition on the antioxidant response of kidney are poorly studied in young normotensive and hypertensive rats. The second inhibitor, 7-NI, belongs to a group of indazoles and acts as a selective nNOS inhibitor at certain concentrations. Although chronic 7-NI administration usually does not have direct modulating effects on blood pressure, the proven high nNOS expression in the renal cortex indicates a key regulatory role of NO/nNOS in the kidney in blood pressure regulation. Administration of 7-NI results in glomerular damage, proteinuria, and overall renal deterioration in young SHR [26]. The neuroprotective effect of 7-NI was observed in young normotensive rats, where the antioxidant response of SOD enzymes was stimulated in the brain stem, but not in the L-NAME group. Our previous study suggests that the effect of NOS inhibition is age-dependent [21].

We aimed to determine the effect of chronic inhibition of NOS on the oxidative state and antioxidant response in the renal cortex of normotensive and spontaneously hypertensive rats, where these mechanisms are not clear. Furthermore, we evaluated the effect of nonspecific (total) inhibition of NOS and the importance of nNOS activity in the redox processes.

## 2. Materials and Methods

### 2.1. Chemicals

Most of the chemicals and reagents were obtained from Sigma-Aldrich (Saint Louis, MO, USA); when not, the company is indicated.

### 2.2. Guide for the Use and Care of Laboratory Animals

Procedures were performed in accordance with institutional guidelines and were approved by the State Veterinary and Food Administration of the Slovak Republic and by the Committee on the Ethics of Procedures in Animal, Clinical and other Biomedical Experiments (permit number: EK/noh2s/14) of the Institute of Normal and Pathological Physiology, Centre of Experimental Medicine, Slovak Academy of Sciences, according to the European Convention for the Protection of Vertebrate Animals used for Experimental and other Scientific Purposes, Directive 2010/63/EU of the European Parliament. All rats used in this study were received from the accredited breeding establishment of the Institute of Normal and Pathological Physiology, Slovak Academy of Sciences (permit number: SK U 14016), and were housed under a 12 h light-12 h dark cycle, at a constant humidity (45–65%) and temperature (20–22°C), with free access to standard laboratory rat chow and drinking water. The Institute of Normal and Pathological Physiology provided veterinary care. The health state of the animals was daily monitored and appreciated according to specific criteria used to determine when animals should be euthanized (human endpoint). The features of the body (weight shortage), physiological functions (food and water intake, disequilibrium, etc.), and social interactions (avoidance reactions, uncoordinated movement, etc.) of the animals were included into these criteria.

### 2.3. Animal Models and Treatments

Young males of normotensive Wistar rats (WRs, age 4 weeks) and spontaneously hypertensive rats (SHRs, age 4 weeks) were used. Young animals were divided into three groups for each strain by the type of administered compounds. The first group of young mice was treated with 7-nitroindazole (7-NI, Sigma-Aldrich) diluted in drinking water at a dose of 10 mg/kg/day (WR+7-NI, *n* = 7; SHR+7-NI, *n* = 9). The second group of young rats was treated with N(G)-nitro-L-arginine-methyl ester (L-NAME, Sigma-Aldrich) diluted in drinking water at a dose of 50 mg/kg/day (WR+L-NAME, *n* = 7; SHR+L-NAME, *n* = 5). The third group of young rats was the control group with pure drinking water (WR, *n* = 7; SHR, *n* = 8). L-NAME is an L-arginine derivative that nonselectively inhibits NOS activity. Administration of L-NAME to young SHRs involves kidney damage, and it is often used as a model for studying chronic kidney diseases typically observed in patients with essential hypertension [27]. Used doses of the inhibitors were adjusted based on previous experiences from our laboratory as well as observations of other experimental works [21, 28, 29]. Experimental studies have observed that higher doses of 7-NI result in nonspecific inhibition of NOS and the inhibition of eNOS has also been observed [30]. Oral administration of NOS inhibitors (L-NAME and 7-NI) used in our study is a commonly used method for studying NO-deficient hypertension. Direct action of the pharmacological agents on the kidney was expected based on chemical ADMET (Absorption, Distribution, Metabolism, Excretion, and Toxicity profiles) properties of 7-nitroindazole and N(G)-nitro-L-arginine methyl ester. The ADMET structure-activity relationship server (admetSAR) is a comprehensive knowledge base and a tool for predicting ADMET properties of drug candidates [31]. ADMET predicted profile modelled by admetSAR showed that 7-nitroindazole has a high probability (0.9911) of intestinal absorption (IA+). L-NAME has an intestinal absorption probability of 0.6725. Transport mechanisms of L-NAME are not identified, but L-NAME is an analog of L-arginine, which is transported through the cationic amino acid transporter system y+. Amino acid transporters are present in the intestine and the kidney [32]. These transporters may be one of the possible mechanisms of L-NAME transportation.

Both substances, 7-NI and L-NAME, were administered continuously for up to 6 weeks. The period of treatment in SHR+L-NAME was shortened due to abrupt increased mortality in this group, and rats were sacrificed by decapitation during the fifth week of administration. All efforts were made to minimize the suffering of the animals.

### 2.4. Measurement of Systolic Blood Pressure

Noninvasive plethysmography was used to measure systolic blood pressure on the tail through Statham Pressure Transducer P23XL (Hugo Sachs, Germany) in all groups of rats. Measurement of blood pressure was realized every week at the same time during the whole period of the experiment.

### 2.5. Sample Preparation

After long-term therapy, rats were sacrificed by decapitation after a brief anesthetization with CO_2_. The kidneys were quickly extracted, the renal fibrous capsule was removed, and the renal cortex tissue was stored in ice Tris-HCl with the addition of protease inhibitors for further measurements. The remaining samples were rapidly frozen in liquid nitrogen and stored at -80°C until use. The amount of proteins was determined by the Lowry method.

### 2.6. Determination of NOS Activity

The activity of NOS was measured by conversion of radioactive [3H]-L-arginine (Amersham, UK) to 3H-L-citrulline according to a previous procedure [21]. After the incubation (6 min at 37°C) of pairs of samples and the reaction mixture (consisting of 10 mM NADPH; 0.5 M Tris, pH 7.4; 20 mM CaCl2 (MgCl2); 100 *μ*M L-arginine; 1 mg/ml calmodulin; FAD/FMN 1 : 1; radio-labelled L-arginine; 50 mM BH4; distilled water), the reaction was started by adding the reaction mixture to the samples. A solution with 0.02 M HEPES, 2 mM EDTA, 2 mM EGTA, and 1 mM L-citrulline was used to stop the reaction after 20 min. Samples were applied to a Dowex column in the Na+ cycle, and the product with scintillation fluid ECOLIT was evaluated on a Tri-Carb 2910 TR (PerkinElmer, USA) scintillation counter. The total activity of NOS was expressed as pkat/g of proteins.

### 2.7. Measurement of SOD Activity

Total SOD activity was analysed by the SOD Assay Kit (Sigma-Aldrich, Germany) in 0.5% homogenates in Tris-HCl with the addition of protease inhibitors according to the manufacturer's protocol. The activity of SOD was measured as inhibition of the production of formazan (WST-1-formazan) from tetrazolium salt (WST-1), which reacts with superoxide anions. Samples were incubated for 20 min at 37°C. Absorbance was measured on a spectrophotometer (Thermo Scientific Multiskan FC, USA) at 450 nm. The resulting values were calculated using a standard curve and expressed as U/mg (unit/milligram) of proteins.

### 2.8. Western Blotting

Briefly, samples of the kidney cortex (20 *μ*g total protein) were separated on a 15% polyacrylamide gel by electrophoresis. Separated proteins were transferred from the gel to a nitrocellulose membrane. The membranes were incubated with Ponceau S red stain solution for 10 min to verify good transfer following blocking with Tris-buffered saline+Tween 20 (TBS-T) containing 5% dry milk. After blocking, the membranes were incubated with a primary antibody. The following antibodies were used to detect the protein expression of SOD isoforms: rabbit polyclonal anti-superoxide dismutase 3 antibody (Abcam, Cambridge, UK; 1 : 1000 dilution, overnight incubation), rabbit polyclonal AntiSOD2/MnSOD antibody (Abcam, Cambridge, UK; 1 : 2000 dilution, 2 h incubation), and rabbit polyclonal anti-SOD1 antibody (Santa Cruz Biotechnology, USA; 1 : 2000 dilution, 2 h incubation). To detect the protein expression of 3-nitrotyrosine, a mouse monoclonal anti-3-nitrotyrosine antibody [11C2] (Abcam, Cambridge, UK; 1 : 100 dilution, overnight incubation) was used. Following three washes (3 × 10 min) with TBT-T, the membranes were incubated for 1 h with horseradish peroxidase labelling antibody (anti-rabbit IgG HRP-linked antibody, 1 : 2000 dilution, Cell Signaling Technology, MA, USA; stabilized anti-mouse IgG, (H+L) peroxidase conjugated antibody, 1 : 1000 dilution, Thermo Fisher Scientific, MA, USA). Primary and secondary antibodies were diluted in TBS-T containing 1% dry milk. As an internal control, all blots were reprobed with GAPDH. We used a mouse monoclonal anti-GAPDH antibody (Santa Cruz Biotechnology, USA; 1 : 2000 dilution, overnight incubation). Enhanced luminol-based detection (Amersham ECL Prime Western Blotting Detection Reagent, GE Healthcare Life Sciences, UK) was used for visualization of bands using ChemiDoc™ Touch Imaging System (Bio-Rad, Hercules, CA, USA) and quantified by Image Lab Software as volume (intensity), normalized to the housekeeping protein GAPDH to correct for variations in total protein loading and for an internal standard. Protein abundance was represented as protein/GAPDH (A.U. (arbitrary units)).

### 2.9. Statistical Analysis

The data are expressed as the mean ± SEM. The effect of inhibitors on the biometric parameters and biochemical and immunoblot analysis in Wistar and SHR was analysed using two-way ANOVA (strain × inhibitors) with Bonferroni's *post hoc* test. To evaluate the changes in systolic blood pressure, three-way ANOVA (strain × inhibitors × duration) was used with Bonferroni's *post hoc* test. Differences between means were considered significant at *P* < 0.05. For statistical analysis, OriginLab software was used (OriginLab Corporation, MA, USA).

## 3. Results

Chronic 7-NI and L-NAME treatment did not affect the general biometric parameters in WRs (Table 1). On the other hand, we observed unexpectedly increased mortality in the SHR group treated with L-NAME (37.5%) during 5 weeks of treatment. Body weight was significantly decreased in the SHR+L-NAME group compared to the SHR (^a^*P* < 0.05) and SHR-7NI (^a^*P* < 0.05) groups, and SHR animals had decreased body weight compared to normotensive rats (^∗^*P* < 0.05). Body weight was differently affected by the strain of rats (*F*_(1, 41)_ = 564.711; *P* = 1.35 × 10^−23^) and the type of inhibitor used (*F*_(1, 41)_ = 12.226; *P* = 8.87 × 10^−5^). Moreover, there was a significant effect of strain-inhibitor interaction (*F*_(1, 41)_ = 11.617; *P* = 1.28 × 10^−4^) (Table 1). In SHR groups, the weight of the kidney was decreased compared to normotensive groups (^∗^*P* < 0.05). There was a significant effect of the strain (*F*_(1, 34)_ = 90.6; *P* = 2 × 10^−10^) and inhibitor (*F*_(1, 34)_ = 3.621; *P* = 0.039) interaction. We observed an increase in the ratio of kidney weight to body weight in SHR+L-NAME compared to control SHR group (^a^*P* < 0.05) and SHR+7-NI (^a^*P* < 0.05). This increase was probably caused by a decrease in the body weight of rats in this group. SHR had decreased the ratio of kidney weight to body weight compared to normotensive rats (^∗^*P* < 0.05). Moreover, there was a significant effect of the strain (*F*_(1, 34)_ = 298.667; *P* = 8.26 × 10^−17^) and the inhibitors (*F*_(1, 34)_ = 23.587; *P* = 8.29 × 10^−7^) and strain-inhibitor interaction (*F*_(1, 34)_ = 21.623; *P* = 1.79 × 10^−6^) (Table 1).

**Figure 1:**
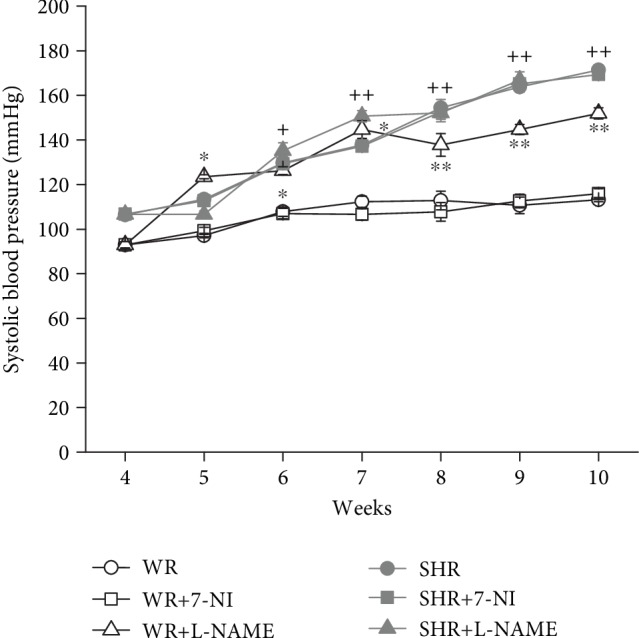
Systolic blood pressure (mmHg) in Wistar (WR) and spontaneously hypertensive rats (SHR) after chronic inhibition of NOS. Data show the mean ± sem. ^∗^*P* < 0.05 and ^∗∗^*P* < 0.01 WR+L-NAME compared to WR or WR+7-NI; ^+^*P* < 0.05 and ^++^*P* < 0.01 SHR compared to WR. L-NAME: N(G)-nitro-L-arginine-methyl ester; 7-NI: 7-nitroindazole.

The values of the systolic blood pressure (SBP) increased with the weeks (*F*_(4, 297)_ = 75.89; *P* = 0). There was a strain-duration (*F*_(4, 297)_ = 34.16; *P* = 0) and treatment-duration (*F*_(4, 297)_ = 3.57; *P* = 1.91 × 10^−4^) interaction in the measured values of SBP. In young WR, SBP has risen only after L-NAME treatment compared with the WR and WR+7-NI groups (Figure 1^∗^*P* < 0.05). In general, SHRs had a significantly increased SBP compared with WR; however, in SHR, the NOS inhibition (neither by 7-NI nor by L-NAME) did not affect the SBP in this strain.

**Figure 2:**
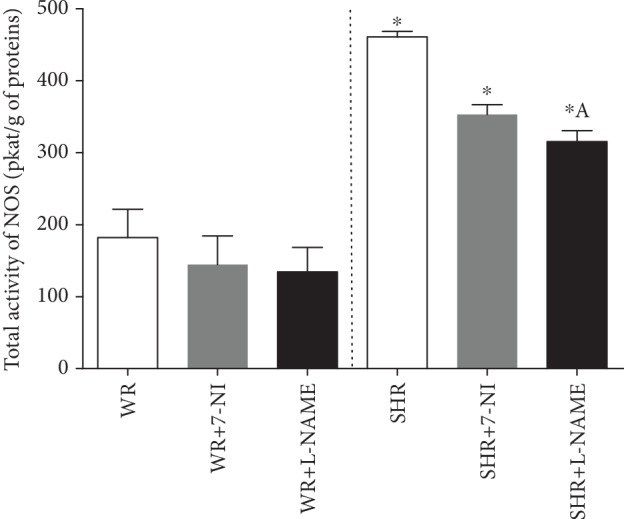
Total activity of NOS in the renal cortex of young Wistar (WR) and spontaneously hypertensive rats (SHR). Data show the mean ± sem. ^∗^*P* < 0.05 SHR groups compared to WR groups; ^A^*P* < 0.05 SHR+L-NAME compared to SHR. L-NAME: N(G)-nitro-L-arginine-methyl ester; 7-NI: 7-nitroindazole.

NOS inhibitors (*F*_(1, 37)_ = 6.823; *P* = 0.0034) and the strain (*F*_(1, 37)_ = 101.543; *P* = 1.88 × 10^−11^) had impacts on the total activity of NOS in the renal cortex. While NOS activity was decreased after treatment with L-NAME in SHR compared to the control SHR group (^a^*P* < 0.05), any inhibitory effect of 7-NI or L-NAME was observed in the kidneys of Wistar rats. Hypertensive animals had elevated total activity of NOS in the renal cortex compared to normotensive rats (Figure 2).

**Figure 3:**
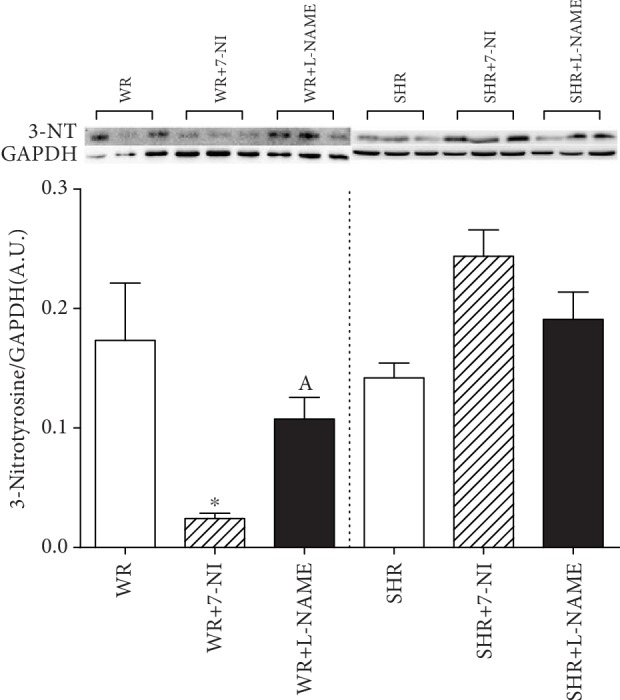
Protein expression of 3-nitrotyrosine in the renal cortex of young Wistar (WR) and spontaneously hypertensive rats (SHR). Data show the mean ± sem. ^∗^*P* < 0.05 WR+7-NI compared to WR, SHR, SHR+L-NAME, and SHR+7-NI; ^A^*P* < 0.05 WR+L-NAME compared to SHR+7-NI. L-NAME: N(G)-nitro-L-arginine-methyl ester; 7-NI: 7-nitroindazole.

3-NT is the most commonly used peroxynitrite biomarker in biological systems. Inhibition of nNOS had the opposite effect on nitrotyrosine immunoreactivity in Western blot analysis depending on the strain of rat (*F*_(1, 48)_ = 19.410; *P* = 6.89 × 10^−5^). Chronic administration of 7-NI led to a reduction of 3-NT production in WRs (^∗^*P* < 0.05, WR+7-NI vs. WR) and an upward trend in SHRs. Moreover, there was an observed significant effect of strain-inhibitor interaction (*F*_(1, 48)_ = 12.235; *P* = 6.22 × 10^−5^) in expression of 3-NT (Figure 3).

**Figure 4:**
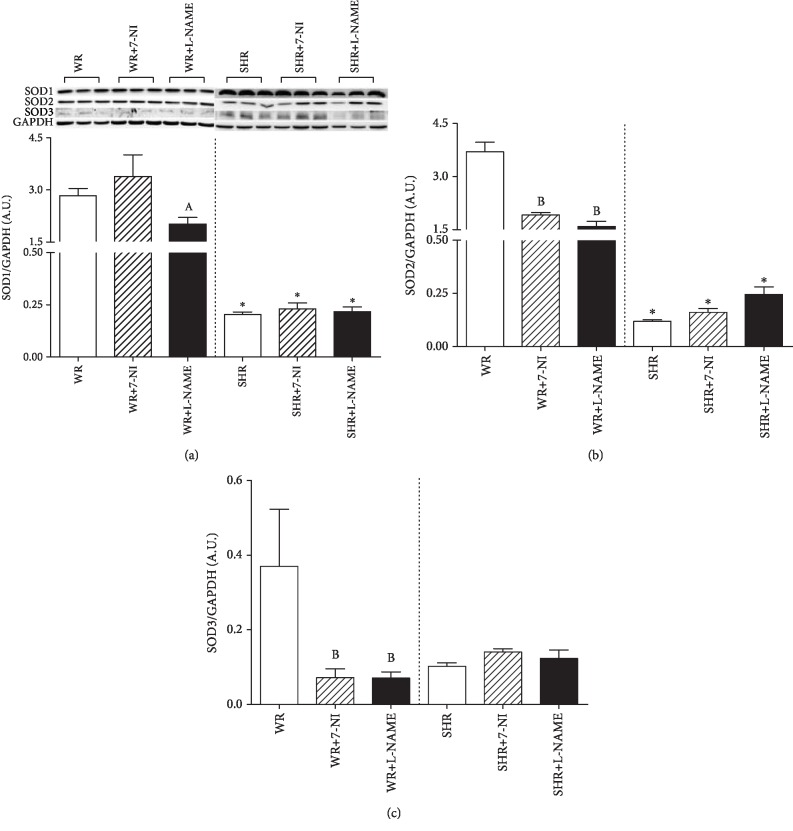
Protein expression of SOD isoforms in the renal cortex of Wistar (WR) and spontaneously hypertensive (SHR) rats. Data show the mean ± sem. ^∗^*P* < 0.05 SHR groups compared to WR groups; ^A^*P* < 0.05 WR+7-NI compared to WR+L-NAME; ^B^*P* < 0.05 compared to control WR. L-NAME: N(G)-nitro-L-arginine-methyl ester; 7-NI: 7-nitroindazole.

Immunoblotting analyses showed different effects of inhibitors in normotensive Wistar rats and SHRs on the protein expression of individual isoforms of SOD. SOD1 expression was significantly decreased only after L-NAME inhibitor in WR compared to the WR+7-NI group (^a^*P* < 0.05, Figure 4(a)). There was no effect on the expression of SOD1 in SHRs after NOS inhibition, but a significant effect of the strain was observed (*F*_(1, 56)_ = 87.594; *P* = 1.17 × 10^−12^). SHR groups have significantly lower protein expression of SOD1 compared to WR groups (Figure 1(a); ^∗^*P* < 0.05). The expression of SOD2 (^a^*P* < 0.05, Figure 4(b)) and SOD3 (^a^*P* < 0.05, Figure 4(c)) was significantly reduced in normotensive WR after both 7-NI and L-NAME. In SHRs, the protein expression of SOD2 and SOD3 was lower compared to WRs, but an increasing trend was present for the expression of SOD2 after chronic treatment with L-NAME and for SOD3 expression after chronic inhibition with 7-NI in the SHR. A significant effect of the strain (*F*_(1, 48)_ = 584.575; *P* = 1.17 × 10^−26^) and inhibitors (*F*_(1, 48)_ = 45.066; *P* = 2.8 × 10^−11^) and the strain-inhibitor interaction (*F*_(1, 48)_ = 54.297; *P* = 1.72 × 10^−12^) was detected for the expression of SOD2. A significant effect of strain-inhibitor interaction (*F*_(1, 50)_ = 3.961; *P* = 0.026) was present for the expression of SOD3.

**Figure 5:**
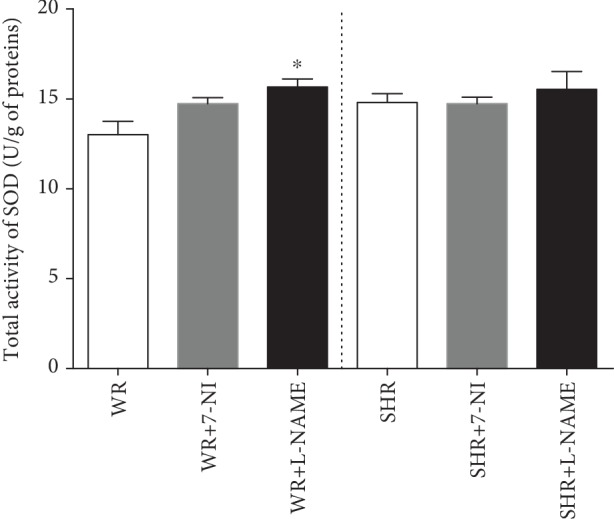
Total SOD activity in the renal cortex of Wistar (WR) and spontaneously hypertensive (SHR) rats. Data show the mean ± sem. ^∗^*P* < 0.05 WR+L-NAME compared to WR. L-NAME: N(G)-nitro-L-arginine-methyl ester; 7-NI: 7-nitroindazole.

The measurement of total SOD activity showed no changes after specific inhibition of nNOS with 7-NI, but an increase was observed after treatment with L-NAME in normotensive WRs (^∗^*P* < 0.05 vs. WRs; Figure 5). There were no differences in total SOD activity after NOS inhibition in SHRs and between normotensive and hypertensive animals (Figure 5). A significant effect of strain-inhibitor interaction (*F*_(1, 50)_ = 3.961; *P* = 0.026) was detected.

## 4. Discussion

In the present study, we determined how chronic inhibition of constitutive NOS isoforms affects oxidative state and antioxidant response in the renal cortex of young normotensive and spontaneously hypertensive rats. We evaluated the differences in the NO signaling pathway and antioxidant defense and their impact on oxidative status in the kidney.

L-NAME is a nonspecific inhibitor of NOS enzymes that is often used for studying the role of NO in different systems involved in the regulation of blood pressure. Basal blockade of NOS with L-NAME leading to elevation of blood pressure (BP) causes hypertension and systemic and renal vasoconstriction in adult normotensive rats [16]. In keeping with previous findings in adult rats, regular blood pressure measurements during the inhibition period in our study have shown increased SBP in young WRs after L-NAME treatment. Similar to normotensive rats, in young SHRs, chronic inhibition of NOS with L-NAME may lead to increased BP [25]. We observed a decrease in BP in the SHR+L-NAME group after 1 week and a rapid increase in BP from 100 mmHg to 150 mmHg over the following two weeks of L-NAME administration. However, BP did not increase further after 4 weeks of L-NAME administration compared to the control SHR group. Such a rapid increase in blood pressure could lead to tissue and organ pressure damage and the associated increased mortality in this group. Selective inhibition of nNOS with 7-nitroindazole did not change the BP in normotensive and SHRs [33, 34], whereas according to Ollerstam et al. [35], BP increased in adult Sprague Dawley rats after chronic inhibition of nNOS. In the present study, we observed that young rats were sensitive to chronic inhibition of constitutive isoforms of NOS with L-NAME, but specific inhibition of nNOS with 7-NI did not influence BP in young Wistar rats or SHR. The weakened effect of L-NAME on BP in young SHR is probably due to the increased ability to adapt to the reduced bioavailability of NO by higher NO synthase activity compared to normotensive Wistar rats [28], and higher expression of NOS isoforms was observed in SHR compared to Wistar Kyoto rats [36, 37]. Moreover, although we showed that the chronic inhibition of NO levels led to negative and malignant effects in young SHR, Berenyiova et al. [28] demonstrated no crucial decrease in NO levels in isolated arteries despite chronic treatment with L-NAME.

Acute or chronic inhibition of NO with L-arginine analogues is associated with renal functional and structural alterations. In addition, the long-term administration of L-NAME results in hypertension, leads to tubular and glomerular lesions, and reduces renal function [15, 16, 38, 39]. The ratio of kidney weight to body weight is a commonly used parameter in the study of hypertensive-related diseases because it can demonstrate various pathophysiological changes in the tissue, including hypertrophy of different kidney structures. We did not observe changes in the kidney weight to body weight ratio after chronic inhibition of NO (with 7-NI or L-NAME) in Wistar rats, but we did not examine structural changes in detail. In SHR, hypertension develops during the first 10 weeks after birth, but hypertensive kidney damage is not morphologically evident [40]. The model SHR+L-NAME is often used to study chronic kidney disease and is accompanied by pathophysiological changes, such as glomerulosclerosis and nephrosclerosis [27]. In our study, inhibition of nNOS did not alter the kidney weight to body weight ratio in SHRs. In accordance with our results, Huang et al. [26] did not observe changes in kidney weight after chronic inhibition with 7-NI but showed impairment of renal function, proteinuria, and glomerular damage, indicating a significant renoprotective role of nNOS in the kidney of SHR. Similarly, studies did not observe changes in kidney weight in SHR after L-NAME administration, despite functional and structural renal impairment [25, 41]. Our biometric parameters show that the kidney weight to body weight ratio was greater in SHR+L-NAME, but this enhancement was caused by lower body weight in this group. Similarly, a decrease in body weight was observed by Cheng et al. [25] in young SHRs after L-NAME treatment.

Many studies have observed that chronic inhibition of NOS induced with L-NAME successfully decreases the activity of NOS in major systems involved in the regulation of blood pressure, such as the brain, vessels, heart, or kidney, in normotensive and hypertensive rats [21, 42, 43]. However, we did not observe this inhibitory effect in the kidneys of Wistar rats. One possible reason is the age of the rats used in our study because in the abovementioned studies, adult rats were used where renal function decreased with age. The abundance of the substrate, L-arginine, is well-maintained during aging, but there are increases in the concentration of circulating endogenous NOS inhibitors, which could contribute to a powerful inhibitory effect in adult normotensive animals and weakened in young animals [44]. In the renal cortex of normotensive rats, both endothelial and neuronal NOS [45] are abundantly expressed. All three isoforms of NOS (nNOS, iNOS, and eNOS) could have a higher expression of the mRNA or protein level in SHR compared to Wistar Kyoto rats [36, 37]. Unchanged protein expression of eNOS and nNOS was observed after chronic administration of 7-NI or L-NAME in the kidney of young SHR [25, 26]. In our study, the total activity of NOS was decreased after 7-NI or L-NAME in the renal cortex of SHRs, which indicates altered NO signaling in the kidney; this result was not in keeping with findings obtained in normotensive Wistar rats. One possible mechanism leading to discrepancies in NO signaling between WRs and SHRs is the DDAH-ADMA pathway. ADMA (asymmetric dimethylarginine) is an endogenous inhibitor of NOS that is upregulated in hypertensive animals. The accumulation of ADMA in the kidney can be caused by decreased DDAH activity caused by oxidative stress, which is increased in hypertensive rats. Similarly, Cheng et al. [25] showed that chronic treatment with L-NAME significantly decreased DDAH activity and increased oxidative damage in the kidney in young SHRs, which can lead to a decline in total NOS activity. Similarly, Ichihara et al. [46] shows that superoxide anion inhibits the control of afferent arteriolar diameters by nNOS in SHR. Because the specific inhibition of nNOS also affected the total NOS activity, our findings confirm that both the endothelial and the neuronal NOS isoform play significant roles in the kidney of the SHR and that ADMA could interact substantially with NO derived from nNOS.

NO can rapidly react with electron-accepting species, such as oxygen or superoxide, and overproduction of NO and superoxide anions can cause the generation of peroxynitrite, which may be responsible for altering protein function [3, 47]. Nitrated tyrosine is expressed in the renal cortex both in normotensive and in hypertensive rats. The existence of nitration of tyrosine residues on proteins in the kidney in normal circumstances suggests that protein nitrosylation by NO oxidative products may be a normal occurrence related to tonic production of superoxide [48]. This finding is consistent with our results because 3-NT was also expressed in the renal cortex of young Wistar rats. Our previous study observed a neuroprotective effect of 7-NI in young normotensive rats, where the antioxidant response of SOD enzymes was stimulated but not in the L-NAME group [21]. In the present study, we found that 7-NI significantly reduced protein tyrosine nitration and did not affect SOD activity in the renal cortex of WR, suggesting that 7-NI has a protective effect against oxidative stress in normotensive rats, but this effect was not observed after the nonspecific inhibition of NOS with L-NAME. Therefore, the effect of 7-NI is probably not related to nNOS inhibition and SOD antioxidants but is related to the nonspecific effect of inhibitor, and further studies are needed. Studies have shown that chronic inhibition of NOS with L-NAME can lead to oxidative injury of the kidney, which is probably mediated by Ang II in adult normotensive rats [23, 24]. In our study, we did not observe oxidative damage in the kidneys of young Wistar rats after L-NAME administration, which could be related to the increased SOD activity and the stimulated antioxidant defense response observed in this group. In SHR, renal cortical nitrotyrosine deposition is increased two-fold, indicating enhanced interaction of NO with ROS [49]. In our study, we showed that specific inhibition of nNOS and nonspecific inhibition of NOS has stimulating effects on the nitration of proteins in the renal cortex of young SHR. A study of Huang et al. [26] is consistent with our results because 7-NI results in kidney damage in young SHR. Similarly, Cheng et al. [25] observed elevated oxidative damage in the kidney using immunohistochemical staining after administration of L-NAME to young SHR. It seems that inhibition of NOS in SHR could increase the production of ROS followed by increased oxidative renal damage due to a disturbed antioxidant response, which was observed. Heightened expression of 3-NT after both NOS-nonspecific inhibition and nNOS-specific inhibition suggest that nNOS/NO could be involved in kidney protection against the development of oxidative kidney damage in SHRs. In addition, inhibition of nNOS led to impairment of renal function, proteinuria, and glomerular damage in young SHRs [26]. Increased RAS activity and oxidative stress are related and present in SHRs [9, 26, 50]. ROS production can be stimulated via the AT1R-NADPH oxidase pathway in multiple organs [6, 21]. Nox is the central source of ROS in the hypertensive kidney, but a disturbed antioxidant system can also play a role [13, 51]. The previously mentioned factors can be accompanied by an increase in renal cortical nitrotyrosine deposition in SHRs [37] and can, together with differences in NO signaling, affect the extent of oxidative damage in the kidney, which is not in keeping with findings obtained in normotensive Wistar rats.

In the present study, we also evaluated the effect of chronic NOS inhibition on changes in the first line of antioxidant defense—superoxide dismutases (SOD). Three isoforms of SOD are major endogenous antioxidants and are expressed in the kidney [52]. During oxidative stress, generated peroxynitrite can nitrosate and inactivate SOD1 [53], but we did not observe an association between 3-nitrotyrosine and the protein expression of SOD1 in the WR or SHR renal cortex. High levels of ROS in cells and tissues may act as a signal to enhance the activity and expression of antioxidant enzymes. Studies show that chronic L-NAME-induced inhibition of NOS decreases the activity of SOD and glutathione peroxidase but stimulates catalase activity in the kidneys of adult normotensive rats [23, 24]. The results presented in this study with young WR showed that the chronic inhibition of NOS had an attenuated effect on the expression of SOD2 and SOD3 in the renal cortex of young WR, but the total activity of SOD was increased after nonspecific inhibition of NOS with L-NAME. Decreased SOD protein expression may occur as a result of elevated SOD activity, which can be stimulated by NO inhibition. Our results suggest that the functioning antioxidant system of SOD seeks to ensure sufficient bioavailability of NO, which is in keeping with unchanged protein nitration in the kidney cortex in normotensive WR. In the SHR groups, administration of both inhibitors led to increased immunodetection of 3-nitrotyrosine on proteins followed by stimulation of protein expression of SOD2 after L-NAME and SOD3 after 7-NI without changing the total activity of SOD. Different levels of stimulation of SOD isoforms in SHRs after 7-NI and L-NAME may indicate a distinct location of increased production of reactive oxygen species. While SOD2 eliminates superoxide anions in mitochondria, SOD3 protects extracellular NO [12]. Supplementary inhibition of NOS could facilitate efforts to stimulate the antioxidant response through SOD protein expression and attempts to influence SOD activity and increase NO availability. However, impaired antioxidant defense is often observed in SHRs, which is in line with our observations, as the antioxidant activity of SOD was not elevated despite raising SOD protein expression. As a result, increased ROS formation and subsequent protein nitration may have occurred.

## 5. Conclusions

This study highlights the impact of various NO signaling pathways on the antioxidant response and oxidative state in the kidney cortex of normotensive and hypertensive rats. Our experiments demonstrate that both the various NO signaling pathways in young Wistar rats and SHRs and the functionality of the antioxidant defense system play highly important roles in the development of kidney oxidative injury during NO deficiency. It seems that while functioning antioxidant defense system helps to maintain the balance of oxidative state and attempts to dampen the effects of NO deficiency in the renal cortex of WR, the impaired antioxidant response together with NO deficiency could lead to the development of oxidative damage of proteins in the kidney caused by peroxynitrite in SHRs.

## Figures and Tables

**Table 1:** Biometric functional parameters after chronic inhibition of NOS in Wistar rats and SHR.

	WR	WR+7NI	WR+L-NAME	SHR	SHR+7-NI	SHR+L-NAME
Mortality (%)	0	0	0	0	0	37.5
Body weight (g)	388 ± 10	409 ± 12	389 ± 13	248 ± 10^∗^	257 ± 5^∗^	180 ± 9^∗^^a^
Weight of kidney (mg)	2365 ± 104	2597 ± 118	2450 ± 80	1830 ± 75^∗^	1890 ± 37^∗^	1593 ± 100^∗^
Kidney/body weight (mg/g)	5.97 ± 0.17	6.06 ± 0.12	6.06 ± 0.13	7.37 ± 0.1^∗^	7.36 ± 0.08^∗^	8.82 ± 0.15^∗^^a^

Data show the mean ± sem. 7-NI: 7-nitroindazole; L-NAME: N(G)-nitro-L-arginine-methyl ester; WR: Wistar rats; SHR: spontaneously hypertensive rats; ^∗^*P* < 0.05 SHR groups vs. WR groups; ^a^*P* < 0.05 SHR+L-NAME compared to SHR or SHR+7-NI.

## Data Availability

All data arising from this study are contained within the article, and any additional data sharing will be considered by the first author upon request.
